# Broadband noise masks suppress neural responses to narrowband stimuli

**DOI:** 10.3389/fpsyg.2014.00763

**Published:** 2014-07-15

**Authors:** Daniel H. Baker, Greta Vilidaitė

**Affiliations:** Department of Psychology, University of YorkYork, UK

**Keywords:** noise masking, steady-state EEG, suppression, gain control, internal variability

## Abstract

White pixel noise is widely used to estimate the level of internal noise in a system by injecting external variance into the detecting mechanism. Recent work (Baker and Meese, 2012) has provided psychophysical evidence that such noise masks might also cause suppression that could invalidate estimates of internal noise. Here we measure neural population responses directly, using steady-state visual evoked potentials, elicited by target stimuli embedded in different mask types. Sinusoidal target gratings of 1 c/deg flickered at 5 Hz, and were shown in isolation, or with superimposed orthogonal grating masks or 2D white noise masks, flickering at 7 Hz. Compared with responses to a blank screen, the Fourier amplitude at the target frequency increased monotonically as a function of target contrast when no mask was present. Both orthogonal and white noise masks caused rightward shifts of the contrast response function, providing evidence of contrast gain control suppression. We also calculated within-observer amplitude variance across trials. This increased in proportion to the target response, implying signal-dependent (i.e., multiplicative) noise at the system level, the implications of which we discuss for behavioral tasks. This measure of variance was reduced by both mask types, consistent with the changes in mean target response. An alternative variety of noise, which we term zero-dimensional noise, involves trial-by-trial jittering of the target contrast. This type of noise produced no gain control suppression, and increased the amplitude variance across trials.

## INTRODUCTION

Physical implementations of signal transduction systems suffer from degraded information transmission owing to internal noise. This is true both for electronic systems, such as amplifiers, and for biological sensory systems like the human visual system. It is of substantial interest to the study of basic perceptual processes (Kersten, 1984; Pelli, 1985; Legge et al., 1987; Gold et al., 1999; Allard and Faubert, 2006; Goris et al., 2008; Hess et al., 2008; Lu and Dosher, 2008; Baker and Meese, 2012), as well as clinical disorders (Pardhan et al., 1996; Levi and Klein, 2003; Pelli et al., 2004; Sperling et al., 2005; Xu et al., 2006; Huang et al., 2007; Milne, 2011), to be able to estimate the magnitude of this internal variability.

The standard method for estimating internal noise is to assess how task performance degrades in varying levels of external noise (Pelli, 1981; Lu and Dosher, 2008). The external noise will introduce variance into the detecting channel and raise thresholds for (e.g., reduce sensitivity to) target stimuli by decreasing the signal-to-noise ratio (see Appendix 1). The external noise level at which performance starts to become poorer is referred to as the “equivalent internal noise,” as it is the point at which the external noise is equal in magnitude to the internal noise. Various techniques exist for estimating this value, including fitting computational models (Lu and Dosher, 2008) and using Bayesian adaptive methods (Lesmes et al., 2006).

However, it has long been appreciated (Watson et al., 1997) that broadband white noise masks might have other effects on signal detection besides increasing within-mechanism variance. There are several pieces of evidence that support a more complex account. Firstly, the slope of the psychometric function for contrast detection does not always become linear in noise (Klein and Levi, 2009; Baker and Meese, 2012), as would be predicted by Birdsall’s theorem (Lasley and Cohn, 1981) for an individual nonlinear channel being swamped by external variance. Furthermore, the consistency of observer responses in noise across multiple passes through an identical trial sequence is lower for broadband noise than would be expected based on its masking potency (Burgess and Colborne, 1988; Lu and Dosher, 2008; Baker and Meese, 2012). Lastly, strong masking effects are observed even when the same sample of noise is used in both trial intervals (Watson et al., 1997; Beard and Ahumada, 1999; Baker and Meese, 2012); a result that would not occur for a noisy ideal observer limited only by variance.

What might be responsible for these deviations from the performance expected due to increased variance in the detecting channel? A plausible candidate is contrast gain control suppression (Heeger, 1992; Carandini and Heeger, 1994; Foley, 1994; Tolhurst and Heeger, 1997; Freeman et al., 2002; Sit et al., 2009; Carandini and Heeger, 2012) of the detecting mechanism by nearby mechanisms sensitive to other orientations and spatial frequencies also present in the noise mask. Several recent studies (Baker and Meese, 2012, 2013; Hansen and Hess, 2012) have provided behavioral evidence that supports this hypothesis. However, the possibility still remains that other processes, such as induced uncertainty or induced internal noise (Lu and Dosher, 2008), might be involved. The present study used the steady-state visual evoked potential (SSVEP) technique (e.g., Tsai et al., 2012) to measure the neural response to contrast directly at the scalp. We show that broadband white noise masks have a powerful suppressive effect (see also Skoczenski and Norcia, 1998), very similar to that of narrowband orthogonal grating masks.

## MATERIALS AND METHODS

Stimuli were displayed on a gamma corrected Iiyama VisionMaster Pro 510 monitor using a Bits# stimulus generator (Cambridge Research Systems, Kent, UK). The monitor had a refresh rate of 75 Hz and a resolution of 1024 × 768 pixels. When viewed from 57 cm, each degree of visual angle subtended 26 pixels on the display.

Target stimuli were patches of sine wave grating at 1 c/deg displayed at one of five Michelson contrasts (4, 8, 16, 32, or 64%), defined as *C_%_* = 100(*L_max_*-*L_min_*)/(*L_max_*+*L_min_*), where *L* is luminance. Contrast is also expressed throughout in logarithmic (dB) units, where *C*_dB_ = 20*log*_10_(*C_%_*). Stimuli increased and decreased in contrast (in linear units) according to a raised sine wave with a frequency of 5 Hz (on/off flicker), but did not reverse in phase. In the orthogonal mask condition, a second grating with a Michelson contrast of 32% was superimposed upon the target at right angles to it, flickering at 7 Hz. In the 2D noise condition, the mask was broadband white noise, low pass filtered at 5 c/deg, with an RMS contrast of 22%, and also flickering at 7 Hz. Note that the effect of the low pass filtering was to ensure that the majority of the noise power was not lost to very high spatial frequencies, where attenuation from the contrast sensitivity function is substantial. The noise remained white for more than two octaves above the target frequency. A new sample of noise was generated for each trial.

In the “0D” (zero dimensional) noise condition (Baker and Meese, 2012) the stimulus contrast was adjusted on a trial-by-trial basis. Contrasts were sampled from a normal distribution (in linear contrast units) with a standard deviation of 5.6% (15 dB) and added to the target contrast. When the total contrast was negative, the stimulus phase inverted. This meant that in practice the mean absolute contrasts of the lowest two target contrast levels increased to 5.6 and 8.4% in the 0D condition. The higher target contrasts were not materially affected by this phase inversion.

**FIGURE 1:**
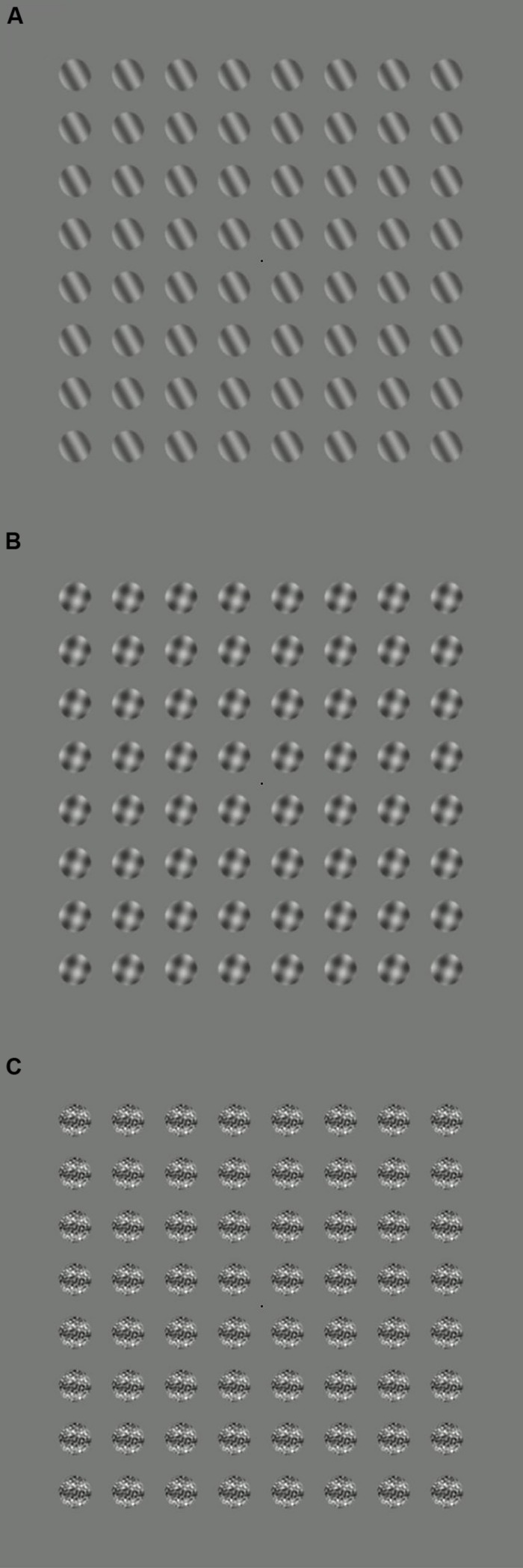
**Example stimuli for three conditions: (A) target only, (B) target plus orthogonal mask, (C) target plus 2D noise mask.** During the experiments, the target stimuli flickered on and off at 5 Hz, and the mask stimuli at 7 Hz.

All stimuli were windowed by a circular raised cosine envelope with a blur width of 4 pixels (0.15°). They were tiled across the monitor in an 8 × 8 grid (see **Figure 1**), and were displayed for trial durations of 11 s. To minimize adaptation, the orientation of the stimulus patches was randomized on every trial. There were five target contrast levels, and five stimulus configurations (no stimulus, target only, orthogonal mask, 2D noise mask, and 0D noise), which combined factorially to give 25 conditions. Observers completed five blocks, in which each condition was repeated twice (10 repetitions in total), taking around 1 h. Six adult observers completed the experiment; all had normal or optically corrected vision.

We recorded EEG signals at 64 electrode locations, distributed across the scalp according to the 10/20 system in a WaveGuard cap (ANT Neuro, Netherlands). We also recorded the vertical electrooculogram using self-adhesive electrodes placed above the eyebrow and at the top of the cheek on the left side of the face. Signals were amplified and then digitized using a PC running the ASA software (ANT Neuro, Netherlands).

The data were imported into Matlab (Mathworks, MA, USA) and analyzed oﬄine. We used average referencing to normalize all waveforms to the mean of all 64 electrodes (at each temporal sample). Each trial was split into eleven one second segments. The first 1s was discarded to eliminate onset transients, and the remaining ten 1 s segments were Fourier transformed, with the phase and amplitudes recorded at both the target and mask frequencies (5 and 7 Hz). These ten observations were combined using coherent averaging to give a single measure of phase and amplitude for each trial at each electrode. We averaged across trials within each observer, and then calculated grand averages and standard errors across observers. The same procedure was used to average the signal variances.

## RESULTS

**FIGURE 2:**
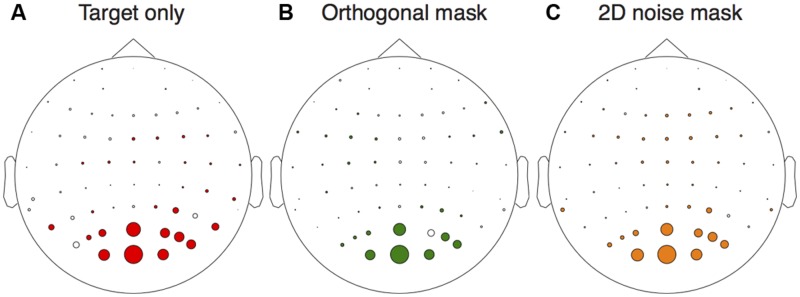
**Electrical activity across the scalp relative to the target absent conditions.** The target only comparison **(A)** was between responses to the highest contrast target (36 dB) and the target absent condition at the target frequency (5 Hz). The orthogonal **(B)** and 2D noise **(C)** mask comparisons were made at the mask frequency (7 Hz) for the lowest contrast target (12 dB). In each panel, the diameter of the circles is proportional the amplitude difference between the conditions tested. Circles are shown in color if this difference was significant (paired *t*-test across observers, *N* = 6) at *p* < 0.05.

We first assessed activity at the stimulus frequencies across the electrode montage. We compared responses at 5 Hz between target absent trials, and trials on which the highest contrast target was shown in isolation. From **Figure 2A** it is clear that the strongest responses (largest colored circles) were observed at occipital electrodes. A similar pattern occurred for activity at 7 Hz when comparing target absent trials with the conditions in which either the orthogonal (**Figure 2B**) or 2D noise (**Figure 2C**) masks were shown along with the lowest contrast target. We therefore averaged waveforms across the two most active electrodes (*Oz* and *POz*) for the remaining analyses.

**FIGURE 3:**
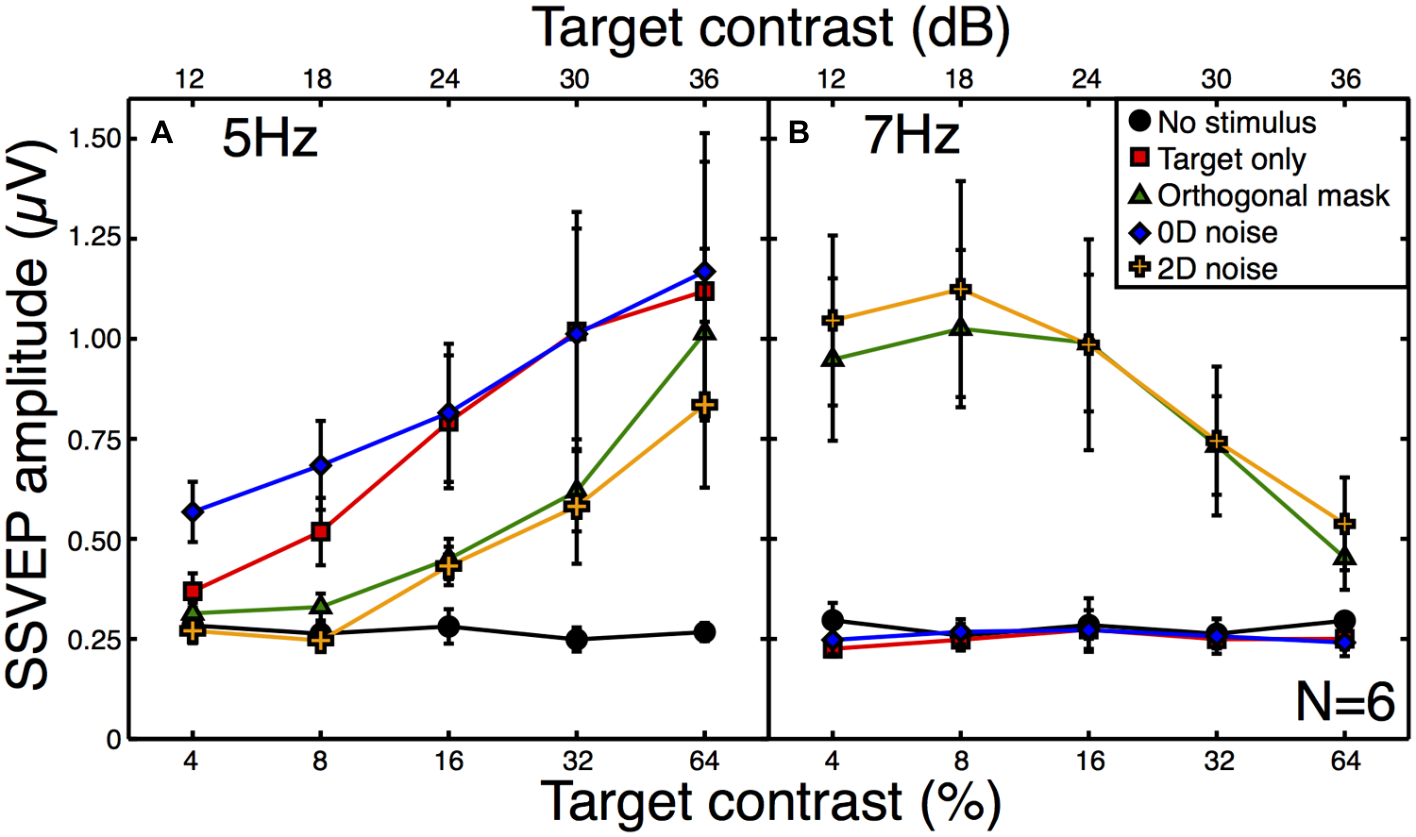
**Steady-state visual evoked potential (SSVEP) amplitudes at the target (A) and mask (B) frequencies.** Amplitude values (with the phase component removed) were averaged across six observers, with error bars giving ±1SE of the mean. The target frequency was 5 Hz and the mask frequency was 7 Hz. Note that for the “no stimulus” condition (black circles), the contrast is irrelevant (all contrasts were 0%), and the five points correspond to five separate repetitions of this baseline condition to illustrate the level of variability in our results.

All observers produced responses that were monotonically increasing functions of target contrast. The average contrast response function to the target alone is shown by the red squares in **Figure 3A**. When a high contrast (30 dB) orthogonal mask was added at a higher temporal frequency (7 Hz), this shifted the contrast response function to the right (green triangles in **Figure 3A**). This is a classic contrast gain control effect, consistent with those reported in previous SSVEP (Brown et al., 1999; Busse et al., 2009; Tsai et al., 2012), fMRI (Brouwer and Heeger, 2011), and neuronal recordings (Morrone et al., 1982; Carandini and Heeger, 1994; Freeman et al., 2002; Busse et al., 2009; Sit et al., 2009).

The broadband white noise mask had a similar suppressive effect on the target response (orange crosses in **Figure 3A**), reducing the amplitude by a slightly greater amount than the orthogonal mask. This rightward shift of the contrast response function (also reported by Skoczenski and Norcia, 1998) is not predicted by standard noisy linear amplifier models of the noise masking process (Pelli, 1981; Lu and Dosher, 2008). There was also a strong response at the mask frequency to both of these masks (**Figure 3B**) which reduced as a function of target contrast. This illustrates the suppressive effects of the target onto the mask (Freeman et al., 2002; Busse et al., 2009; Brouwer and Heeger, 2011; Tsai et al., 2012) and confirms that inhibition occurs in both directions between the neural representations of the stimuli.

By way of comparison, we also measured responses in a 0D masking condition (blue symbols in **Figure 3A**). This involved jittering the stimulus contrast on a trial-by-trial basis. Although this manipulation might appear to make little sense for the single-trial observations of the SSVEP paradigm, it provides a useful comparison with psychophysical data. In 2AFC detection experiments, 0D noise is a very potent mask, raising thresholds by far more than 2D noise (Baker and Meese, 2012). However, it does this without reducing the mean neural response to the stimulus, as shown by the substantial overlap between red and blue functions in **Figure 3A**. The slightly greater response at the two lowest contrasts is easily understood when one considers that for weak target contrasts, large negative jitter values reverse the phase of the stimulus (see Materials and Methods). Since the SSVEP response is invariant with spatial phase it is the *absolute* contrast that determines the response, and this will be slightly higher than the nominal target contrast.

**FIGURE 4:**
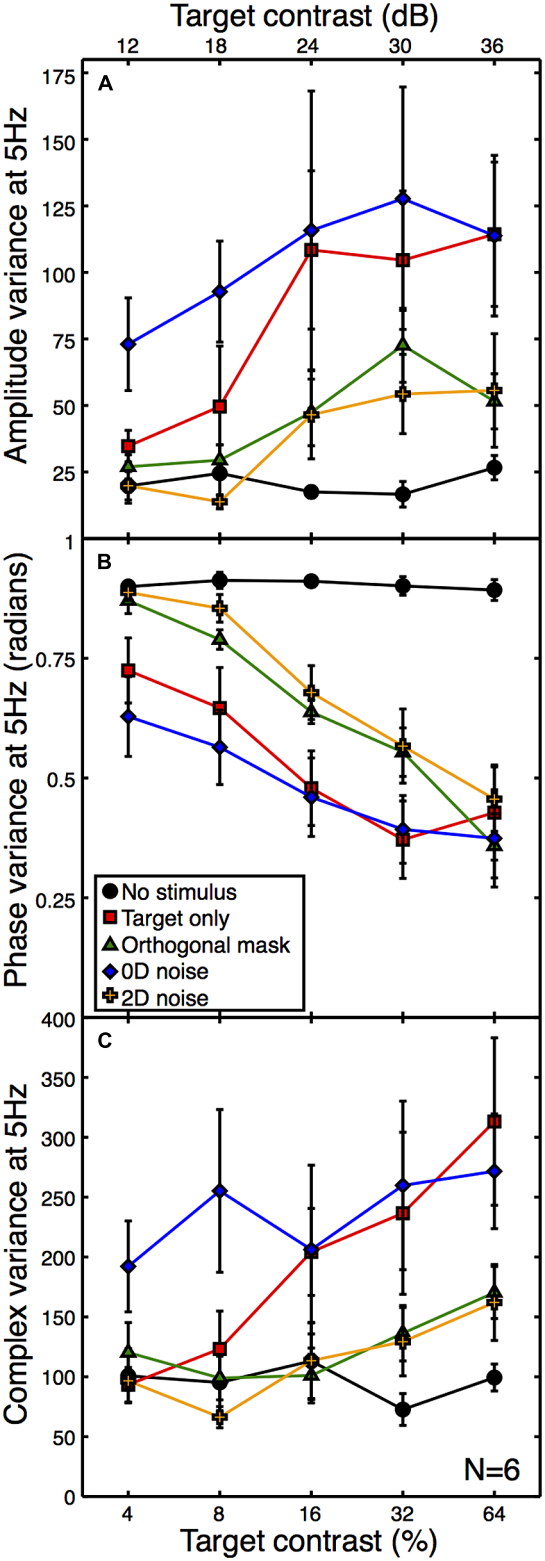
**Mean within-observer variance at 5 Hz as a function of target contrast for the amplitude (A) and phase (B) components, or calculated using the combined (complex) terms (C).** All variances were calculated on a per observer basis and then averaged across observers. The phase data **(B)** were calculated in radians using circular statistics. The phase variance with no stimulus (black) is near the level expected for a set of uniformly distributed random angles. Error bars give ±1SEM.

A second expectation of noise masks is that they increase the variance of neural responses across trials, because each unique noise sample will either increase or decrease activity in the detecting channel by a different amount (see Appendix 1). Note that the error bars in **Figure 3** are not a meaningful index of response variance, as they are calculated across (and not within) observers. To assess response variance, we calculated the trial-by-trial variance within observers for each condition, and then averaged these values across observers (**Figure 4A**). The variances clearly increase as a function of target contrast in all conditions. This is surprising, as it provides direct evidence of signal-dependent (i.e., multiplicative) noise within the visual system (Klein, 2006). The implications of this are discussed below.

One consequence of this signal-dependent noise is that the suppressive effect of the orthogonal mask also reduces the amplitude variance (green functions in **Figure 4A**). A similar reduction in variance is also produced by the 2D noise mask. This is rather worrying, as the aim of using external noise masks is typically to *increase* internal variance, not reduce it! Of course, a consequence of the frequency tagging used in the SSVEP procedure means that a variance increase at the signal frequency is unlikely, but a reduction is truly unexpected. The 0D noise produced an increase in variance at lower target contrasts, but no clear difference at higher target contrasts. This is presumably because the variance of the external noise mask was lower than the signal-dependent internal noise at these target contrast levels.

We also calculated the phase variance at the target frequency using circular statistics. The angular variance in radians was computed across epochs within each observer, and then averaged across observers to produce the plot in **Figure 4B**. High contrast stimuli produced responses that were strongly phase-locked, and so had low trial-by-trial variability. Low contrast stimuli lead to weaker phase locking, so the trial-by-trial variability was higher. The phase variance data in **Figure 4B** reveal a similar arrangement of functions to the other figures, but inverted. This indicates a strong correspondence between signal amplitude and coherence (e.g., the inverse of variance).

Note that as response amplitude increases, the amplitude variance increases but the phase variance decreases. It is therefore unlikely that the greater amplitude variance is a consequence of the phase locking of the SSVEP, as this would predict the opposite direction of effect to the one we report (e.g., amplitude variance would reduce for more coherent responses). However, we also calculated the variance of the complex Fourier components, which includes both phase and amplitude information. These are plotted in **Figure 4C**, and show a similar pattern to the data in **Figure 4A**, suggesting that the two individual variance measures are not confounded.

## DISCUSSION

We measured SSVEPs for patches of sine wave grating in the presence of different types of mask. The contrast response function was shifted rightward by orthogonal grating masks and by broadband noise masks. In addition, these mask types reduced the response variance, which we found to be proportional to the mean response. This pattern of responses suggests that broadband noise has a suppressive gain control effect on the neural response to the target. In comparison, a 0D noise manipulation where the signal strength was varied directly from trial to trial, did not reduce the mean response but did increase the response variance. This is the behavior expected of added external noise (see Appendix 1).

How might the steady-state responses correspond to an observer’s perception, and the decisions they make in perceptual tasks? We make the simplifying assumption that the VEP amplitude at the stimulus temporal frequency is proportional to the total neural population response to that stimulus, and that psychophysical decisions are based on the overall response, rather than the response of a subset of neurons. For contrast detection and discrimination experiments, this seems a reasonable assumption (e.g., Campbell and Maffei, 1970), though we note that it may not hold for more complex tasks (but see Ales et al., 2012). In addition, we made measurements at the occipital pole, which likely reflect activity in early visual areas. Later sources of internal noise could also influence an observer’s decision in perceptual tasks. We note, however, that external noise is likely to have had its primary influence on neural responses by this stage.

Our results support previous misgivings about the ability of broadband noise to appropriately influence an observer’s internal noise (Baker and Meese, 2012). Indeed, the observation that suppression reduces the multiplicative component of internal noise suggests that the problems may be more severe than previously suspected. Future noise masking studies would do well to limit the dimensionality and bandwidth of external noise stimuli as far as possible to mitigate the confounding effect of suppression. The 0D noise stimulus proposed by Baker and Meese (2012) might one way to achieve this aim in some experiments (e.g., Baker and Meese, 2014).

Interestingly, Skoczenski and Norcia (1998) have previously shown that broadband noise masks can shift the contrast response function to the right in both infants and adults. Although they acknowledge that contrast gain control may be responsible for their findings, they analyse their data based on the assumption that the external noise mask increases internal noise multiplicatively (e.g., see Lu and Dosher, 2008). The variance data shown in **Figures 4A,C** is inconsistent with this interpretation, as there is a clear reduction in variance when 2D noise masks are added (at least at the early stages of visual processing that contribute to occipital EEG signals). This speaks against the induced internal noise account of masking (see also Baker and Meese, 2013).

Steady-state VEP techniques are very well established, and have been used in countless studies. Given this ubiquity, we were surprised that previous reports of response-dependent noise were not forthcoming. This may be because the technique has often been used to address developmental (e.g., Skoczenski and Norcia, 1998; Brown et al., 1999) or clinical (e.g., Tsai et al., 2011) issues, rather than as a tool for basic research. We think that the combination of SSVEPs and computational modeling (see particularly Tsai et al., 2012) provides a valuable opportunity to investigate low-level sensory processes such as signal combination and suppression. In the following section, we use a modeling approach to show how the SSVEP data might be linked to psychophysical results.

### RESPONSE-DEPENDENT NOISE: IMPLICATIONS FOR CONTRAST DISCRIMINATION

An unexpected finding was that response variance increases as a function of the mean response. Although this is well established at the level of individual neurons (Tolhurst et al., 1981, 1983), there is evidence that the dominant source of noise at a population level is effectively additive (Chen et al., 2006). In the psychophysics literature, there has been substantial debate over whether noise is additive or multiplicative for behavioral tasks such as contrast discrimination (Kontsevich et al., 2002; Georgeson and Meese, 2006; Klein, 2006; Katkov et al., 2007). Pedestal masking effects (the Weber-like “handle” region of the dipper function) can either be obtained from a nonlinear transducer with additive noise (Legge and Foley, 1980), or a linear transducer with multiplicative noise (Pelli, 1985). Our results suggest that both may be present, since amplitude variance is response dependent (**Figures 4A,C**) and the contrast response function is nonlinear (**Figure 3A**). But which of these two features determines contrast discrimination behavior?

**FIGURE 5:**
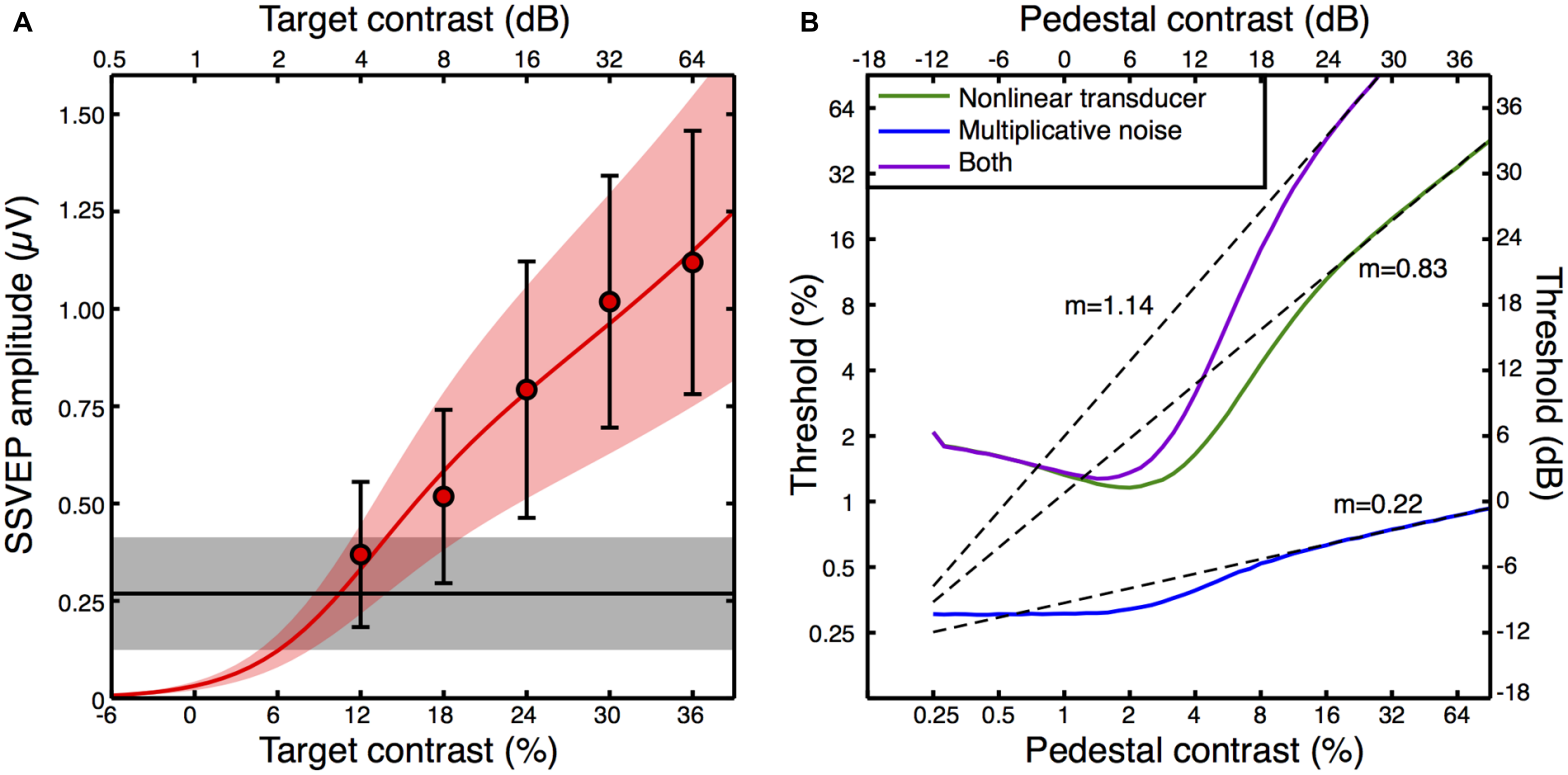
**Transducer model fit to the contrast response function (A), and model predictions for contrast discrimination (B).** The data points in **(A)** are replotted from **Figure 3A**, but with error bars representing the average standard deviation calculated across observers (e.g., the square root of the variances given in **Figure 4A**). The red curve is the best fit of Equation A2 (Appendix 2) with three free parameters, and the orange shaded region indicates the noise standard deviation inferred by fitting Equation A3 (Appendix 2) to the averaged standard deviations (the error bars). The black line is the average response at 5 Hz when no stimulus was shown (the mean of the black symbols in **Figure 3A**), with the gray shaded region giving the standard deviation. The curves in panel **(B)** are simulated contrast discrimination functions based on the fitted parameters. Dashed curves are extrapolated straight line fits to the upper limb of each dipper (pedestal contrasts above 24 dB) using the equation *y* = *mx* + *c* on the dB values. The gradients (*m*) are reported adjacent to each curve.

We fitted a transducer model to the amplitude and variance data from the average contrast response function (see Appendix 2 for details, and **Figure 5A** for the model fit). We then explored the predictions that three variants of this model made for psychophysical contrast discrimination experiments, as shown in **Figure 5B**. In the first variant, we set the multiplicative noise term (Equation A3 in Appendix 2) to zero. The green dipper function therefore shows the prediction based only on the transducer nonlinearity with additive noise. The second variant assumed a linear transducer (*resp* ∝*C*) but with multiplicative noise proportional to the transduced contrast. This model variant, shown by the blue curve in **Figure 5B**, did not feature a dip. Typically facilitation is provided in such models by assuming that intrinsic uncertainty is reduced by the pedestal (Pelli, 1985). However we had no way to constrain such a model using our data set, and our exposition here focusses largely on the rising portion of the dipper. The slopes of the contrast discrimination functions were very different for these two model variants, being 0.83 for the nonlinear transducer and 0.22 for the multiplicative noise model. Finally, we simulated a model that included both a transducer and multiplicative noise. The resulting dipper function (purple curve in **Figure 5B**) had a steeper handle, with a slope of 1.14.

The predicted dipper functions based on our steady state data appear plausible for the nonlinear transducer with additive noise, with a handle gradient somewhat steeper than the slope of ∼0.6 typically reported (Legge and Foley, 1980). When multiplicative noise is added, the handle becomes steeper still, yet even this value of >1 is not inconsistent with previous reports using flickering stimuli similar to ours (Boynton and Foley, 1999). It therefore seems possible that previous attempts to estimate the underlying contrast response function based on psychophysical contrast discrimination data may be inaccurate if they neglect to include a multiplicative noise term. Historically, discrimination performance has been attributed to either a nonlinearity or multiplicative noise (Kontsevich et al., 2002; Georgeson and Meese, 2006; Klein, 2006; Katkov et al., 2007). To our knowledge, this is the first demonstration of how these two factors might combine.

## CONCLUSION

We have presented evidence that broadband noise masks have a suppressive gain control effect on neural responses to narrowband grating stimuli. This effect is similar to that obtained from orthogonal grating masks. Both mask types also reduce the amplitude variance, which is response dependent. We fitted a computational model to the average contrast response function, and used this to infer the relative contribution of a nonlinear transducer and response-dependent noise for contrast discrimination. The modeling indicates that both features may be relevant to psychophysical contrast discrimination performance.

## Conflict of Interest Statement

The authors declare that the research was conducted in the absence of any commercial or financial relationships that could be construed as a potential conflict of interest.

## APPENDIX 1 – ASSUMPTIONS ABOUT NOISE MASKING

A primary tenet of signal detection theory (Green and Swets, 1966) is that performance on a task is determined by the magnitude of a scalar internal response variable. This response is determined by the amplitude of an external signal, and internal variability (noise) within the system. Within the framework of psychophysical “channels” sensitive to a limited range of orientations and spatial frequencies (Blakemore and Campbell, 1969), the internal response in a detection task will be determined by the energy falling within the pass-band of a linear filter, plus internal noise.

When external noise is added to a stimulus, some of the noise power will also fall within the pass-band of the detecting channel. On some presentations, this will increase the mechanism response, whereas on other presentations it will decrease it. Thus, the external noise will introduce variance into the internal response that governs decisions. A formal expression of this process is given by,

(A1) $$resp={\left(C_{test}+\sigma_{ext}R_{1}\right)}^{\gamma }+\sigma_{\mathrm{int}}R_{2},$$

where *C_test_* is the target contrast, *σ_ext_* is the standard deviation of external noise falling within the pass-band of the detecting channel, γ typically has a value of around 2, and σ*_int_* is the standard deviation of internal noise (notation from Klein and Levi, 2009). The terms *R_1_* and *R_2_* denote samples from a Gaussian random number generator that are drawn independently on each trial of an experiment. In this model, the observer bases their decision *only* on the scalar response value; any noise power outside of the detecting channel is ignored.

A number of clear predictions follow from this model that can be tested empirically (e.g., Klein and Levi, 2009; Baker and Meese, 2012). In addition, several elaborations have been proposed that include features such as induced internal noise (Burgess and Colborne, 1988; Lu and Dosher, 2008), gain control suppression (Watson et al., 1997; Baker and Meese, 2012, 2013; Hansen and Hess, 2012) and uncertainty when selecting from multiple channels (Pelli, 1985).

Some studies have designed noise stimuli intended to target a particular stage of processing (Allard and Faubert, 2006; Baker and Meese, 2014), with the aim of increasing the proportion of the noise power that falls within the pass-band of the appropriate detecting mechanism. However, we note that any such manipulations can only influence decision behavior by changing the magnitude of the internal response variable, and so are equivalent to increasing the effective level of the external noise.

## APPENDIX 2 – DETAILS OF CONTRAST TRANSDUCTION MODELS

We fitted a standard transducer nonlinearity (Legge and Foley, 1980) to the target-only contrast response function. The nonlinearity is given by,

(A2) $$resp=R_{\max}\frac{C^{p}}{Z+C^{q}}\,,$$

where *C* is contrast, *p* and *q* are exponents, *Z* determines the gain, and *R_max_* is a scaling parameter. To reduce the number of free parameters, we fixed *q* at the standard value of 2 (Legge and Foley, 1980). We minimized the root-mean-square (RMS) error between the mean amplitude and the model response, and obtained parameter estimates of *p*= 2.24, *Z*= 12.13 and *R_max_*= 4.22. The fit is shown by the red curve in **Figure 5A**.

We then estimated a scaling parameter for multiplicative noise. Since the noise is clearly response-dependent rather than signal dependent (e.g., in **Figures 4A–C** the variances are reduced by the masks), we made the noise proportional to the transducer response,

(A3) $$noise=G_{N\times resp},$$

where *G* represents samples of zero-mean Gaussian noise, with standard deviation determined by the output of Equation A2 (*resp*) and a scaling factor, *N*. We estimated that *N*= 0.35 by finding the value that best described the standard deviations of the responses, shown by the error bars in **Figure 5A** (note that these error bars are the square root of the mean within-observer amplitude variance values given by the red function in **Figure 4A**, and are not the same as the between-observer standard errors in **Figure 3A**). The model noise standard deviation is given by the orange shaded region in **Figure 5A**.

The fitted model parameters were then used to simulate thresholds for contrast discrimination (dipper) functions. To derive predictions at low contrasts, we also required an estimate of fixed (additive) noise. This was obtained from the variance in the target absent condition of the experiment (black symbols in **Figure 4A**). The horizontal black line and gray shaded rectangle in **Figure 5A** show the mean and standard deviation of the 5 Hz amplitude in this condition. We simulated a method of constant stimuli contrast discrimination experiment using the above equations and parameters, with 100,000 stochastic trials per target contrast level. Thresholds were obtained by fitting cumulative Gaussian functions to the simulated data.

The above simulations make several assumptions that may or may not be valid. Least plausible is perhaps our decision to use the variance at 5 Hz in the signal absent condition as an estimate of fixed (additive) internal noise. We think it highly unlikely that the 5 Hz variance when no stimulus is present represents the activity of neurons that subsequently respond to the stimulus, at least in any straightforward way. Many unrelated sources of variance will contribute to this figure, including equipment noise, electromagnetic interference, and spontaneous neural oscillations, so the noise baseline is likely to be an overestimate of the true variance. However, the additive noise term only influences detection and low-contrast discrimination performance (the leftmost parts of the dipper) and has little effect on the slope of the dipper handle. We repeated our simulations for several alternative additive noise levels, and found that the dipper handle gradients remained remarkably constant over a wide range.
